# Comparative assessment of medicine procurement prices in the United Nations Relief and Works Agency for Palestine Refugees in the Near East (UNRWA)

**DOI:** 10.1186/2052-3211-7-13

**Published:** 2014-10-21

**Authors:** Margaret Ewen, Maisa Al Sakit, Rawan Saadeh, Richard Laing, Catherine Vialle-Valentin, Akihiro Seita, Joske Bunders

**Affiliations:** Health Action International, Amsterdam, The Netherlands; Amman, Jordan; UNRWA, Amman, Jordan; Boston University School of Public Health, Boston, MA USA; Harvard Medical School and Harvard Pilgrim Health Care Institute, Boston, MA USA; Athena Institute, Vrij University, Amsterdam, The Netherlands

**Keywords:** UNRWA, Medicines, Procurement, Prices

## Abstract

**Objectives:**

The United Nations Relief and Works Agency for Palestine Refugees in the Near East (UNRWA), the main primary healthcare provider for 4.9 million Palestinian refugees, spent USD18.3 million on essential medicines dispensed free-of-charge through clinics in five areas of operation (‘fields’): Gaza, Jordan, Lebanon, Syria, and the West Bank (2010). Faced with budget contraints and an increasing demand for medicines to treat chronic conditions, the objective of our study was to assess UNRWA’s medicine procurement prices to see if savings could be possible.

**Methods:**

In July 2011, data was collected from UNRWA headquarters in Jordan. Price analyses focused on the top 80 medicines by value, accounting for 93% of pharmaceutical expenditure from the General Fund, with comparisons to international, regional and national references. Prices were also compared for the few medicines procured both through UNRWA’s central tender (centrally) and by the fields directly (locally).

**Results:**

Central procurement prices did not differ markedly from reference prices: median ratios of UNRWA prices to Management Sciences for Health’s International Drug Price Indicator Guide, Jordan’s Joint Procurement Department, Gulf Cooperation Council, and IDA Foundation bulk packs were 0.99, 1.00, 0.98 and 1.12 respectively. Applying the lowest comparator price to five comparatively higher priced medicines would yield savings of USD1.4 million. Local procurements were generally less cost-effective than central tender procurement, with notable differences across fields and medicines.

**Conclusions:**

Overall, UNRWA’s procurement prices were competitive despite the relatively small quantities procured. Regular monitoring of procurement prices and quantities is needed in order to make informed decisions. Our evaluation also underscores the heavy burden of antidiabetic medicines and antimicrobials on UNRWA procurement expenditure.

## Introduction

The United Nations Relief and Works Agency for Palestine Refugees in the Near East (UNRWA), whose headquarters are in Amman Jordan, provides assistance, protection and advocacy for 4.9 million Palestine refugees living in the five areas (‘fields’) of its operation: Jordan, Lebanon, Syria, and the Gaza Strip and West Bank, which are occupied Palestinian territory. Providing primary health care is a key component of this assistance, which has resulted in improvements in the health status of refugees over the last 60 years, in particular in the reduction of maternal and child mortality. This has been achieved despite poor living conditions and high levels of unemployment of refugees, and political instability and frequent conflict in the region.

Medicines are supplied free-of-charge to refugees through 137 primary healthcare centres (PHC) across the five fields, and in one hospital in the West Bank. In 2010, pharmaceutical expenditure was US$18.31 million. Of this, $15.4 million was from UNRWA’s General Fund; the balance is donations and medicines purchased in emergency situations [1]. The top 10 medicines procured in 2010 by value had a total expenditure of US$6.7 million [2]. Of these, three were used to treat diabetes and five were antimicrobials (see Table 1). Expenditure was highest for neutral sol./isophane 30/70 human insulin at approximately $1.5 million (9.6% of General Fund expenditure on medicines). Most medicines are procured via a central open tender, although some medicines are procured by field staff directly. Suppliers are required to be pre-qualified [3]. The lowest bid amidst those technically accepted is awarded the tender (assuming supply and other aspects are satisfactory). Prices are not negotiated although UNRWA can terminate a contract if they can get a lower price. About 85% of awarded contracts are longer term (three years).

**Table 1:** **Top 10 medicines by expenditure in 2010, central and local procurements**

Medicine, strength, dose form	Therapeutic group	Expenditure in USD	Expenditure as % of general fund expenditure on medicines
Neutral sol./isophane 30/70 human insulin 100 IU/ml 10 ml vial	Antidiabetic medicine	$ 1,478,122	9.6%
Amoxicillin + clavulanic acid 500 + 125 mg tab	Antimicrobial	$ 973,521	6.3%
Amoxicillin 250 mg/5 ml suspension	Antimicrobial	$ 716,602	4.7%
Enalapril maleate 10 mg tab	Antihypertensive	$ 661,834	4.3%
Amoxicillin 500 mg cap	Antimicrobial	$ 606,882	4.0%
Amoxicillin + clavulanic acid 250 + 62 mg/5 ml suspension	Antimicrobial	$ 567,409	3.7%
Cephalexin 500 mg cap	Antimicrobial	$ 473,402	3.1%
Metformin HCL 500 mg tab	Antidiabetic medicine	$ 446,482	2.9%
Gliclazide 80 mg tab	Antidiabetic medicine	$ 418,102	2.7%
Paracetamol 250 mg/5 ml suspension	Antipyretic, analgesic	$ 411,496	2.7%
	*Total*	$ 6,753,853	44%

A number of factors are contributing to an increased demand for health care services including an increase in the refugee population, a doubling of the elderly population in the last 30 years, and an epidemiological transition from acute to chronic diseases, in particular diabetes and cardiovascular disease. Chronic diseases account for 70-80% of deaths among refugees, and over the last decade the number of patients with hypertension and/or diabetes increased by 134% [1]. Coupled with increasing demand, UNRWA was facing a funding deficit, estimated to be $63 million in 2011 (from a total budget of $500 million).

Faced with the challenge of providing health services in an environment of growing need and resource constraints, UNRWA initiated a health reform process in 2009 which included various pharmaceutical reviews. Their Essential Medicines List (termed the ‘Rational Drug List’ by UNRWA) was revised in 2010. Reviews of the quantification process, inventory management, and the use of medicines in PHC’s are also included in the reform process.

The objective of our study was to assess UNRWA’s medicine procurement prices and processes. In particular, recent procurement prices would be compared with those from international, regional and national sources, with recommendation on where savings might be possible. The methodology differed from that reported in nine medicine price surveys undertaken in the World Health Organization’s Eastern Mediterranean Region from 2004 to 2006, where only a single comparator was used [4].

The challenges faced by UNRWA in procuring relatively small quantities of medicines in a difficult environment, with differing situations in the five fields where medicines would be delivered, would create an expectation that prices would be higher than in less challenging environments. The price analysis is reported in this article (but not the review of the procurement processes). Findings from both analyses can be found in the report published by UNRWA entitled *Medicine procurement prices and processes in the United Nations Relief and Works Agency for Palestine Refugees in the Near East (UNRWA)* (http://www.unrwa.org/sites/default/files/201210212936.pdf).

## Methods

In July 2011, data was collected from the health, procurement and finance departments at UNRWA headquarters. Prices and quantities procured in 2010, from UNRWA’s General Fund, were extracted from their Procurement Inventory Management System (PIMS) database and analysed [2].

In total 80 medicines were initially selected (out of a total of 143 medicines and vaccines procured), each strength-and dose-form specific, equating to the top 80 medicines by value but excluding a few products more specific to UNRWA (e.g. metacresolsulphonic acid-formaldehyde pessaries), and where comparing products with identical active ingredients and strengths (e.g. multivitamin tablets) could be challenging. The medicines in the analysis accounted for 93% of UNRWA’s pharmaceutical expenditure from the General Fund. The prices included freight and insurance, and any discounts were deducted. For medicines where multiple products were procured, the median price (unweighted) was used.

In the main analysis only 2010 central tender prices were included, thereby reducing the number of medicines from 80 to 77. A separate analysis was undertaken comparing prices for medicines procured in 2010 both through the central tender process (centrally) and those procured by each field (locally). Initially this second analysis was limited to the 80 selected medicines but, as the dataset was small, the analysis was extended to all medicines procured both centrally and locally.

UNRWA’s procurement prices were compared with 2010 prices from Management Sciences for Health’s International Drug Price Indicator Guide (MSH) [5], the Gulf Cooperation Council (GCC) [6], Jordan’s Joint Procurement Department (JPD) [7], and the IDA Foundation (IDA) [8] - see Table 2.

**Table 2:** **Price comparators**

Comparator	Description
Management Sciences for Health’s (MSH) International Drug Price Indicator Guide 2010 http://erc.msh.org	Ex-factory prices offered to low- and middle-income countries by different suppliers. Median supplier prices were used (or median buyer prices where supplier prices were unavailable) plus 15% for freight and insurance.
Jordan’s Joint Procurement Department (JPD) 2010 tender prices http://www.jpd.gov.jo	Pooled procurement for the Ministry of Health, Royal Medical Services, Jordan University Hospital, King Abdullah University Hospital, King Hussein Cancer Centre and the Prince Hamza Hospital. Prices include freight, insurance, any clearance costs and transport to warehouses in Jordan.
Gulf Cooperation Council (GCC) 2010 tender prices	Pooled procurement for the United Arab Emirates, Bahrain, Kingdom of Saudi Arabia, Oman, Qatar Kuwait, and more recently Yemen. Prices include freight, insurance, any clearance costs and transport to warehouses in each country.
IDA Foundation 2010 prices for bulk packs http://www.ida.nl	Not-for-profit supplier of medicines to low- and middle-income countries. Prices include 1.5% handling fee (for orders over Є5000) plus 15% for freight and insurance.

For a few liquid preparations, GCC permits supply within a narrow pack size eg. chlorphenamine 2 mg/5 ml oral solution can be supplied in bottles of 100 - 120 ml. Where the actual pack size supplied was unknown, the analysis was based on the UNRWA procurement unit i.e. 100 ml for chlorphenamine. Comparators prices were excluded in the analysis for pack sizes substantially different to those procured by UNRWA. Prices were included for four medicines where a comparator was found to have a slightly different strength e.g. benzylpencillin benzathine GCC price was for 1MIU/vial whereas the others were 1.2MIU/vial.

Prices were expressed as a ratio of the UNRWA unit price in USD to the comparator unit price in USD. The ratio is thus expression of how much greater or less the UNRWA price is than the comparator price.

Two sensitivity analyses were undertaken to identify potential savings on expenditure based on the quantities procured by UNRWA in 2010 (1) if all matched medicines were procured at JPD, GCC, and IDA prices; and (2) if selected medicines were procured at JPD, GCC, and IDA prices. Three approaches were used to identify individual medicines that offered significant savings potential based on comparator prices: medicines where potential annual savings exceeded US$100,000, UNRWA’s top 10 medicines by expenditure, and antidiabetes medicines.

## Results

There was little difference between UNRWA procurement prices and comparator prices across the medicines where there were matches. Overall UNRWA prices were 1% and 2% lower than MSH and GCC prices respectively, the same as the prices paid by JPD, and 12% higher than IDA bulk prices (see Table 3).

**Table 3:** **Summary of ratios of UNRWA central tender prices to MSH, JPD, GCC and IDA prices**

	MSH	JPD	GCC	IDA
Median ratio UNRWA price to comparator price	0.99	1.00	0.98	1.12
25th percentile ratio	0.66	0.73	0.59	0.76
75th percentile ratio	1.49	1.47	1.39	1.57
Maximum ratio	20.93	2.96	7.55	9.94
Minimum ratio	0.08	0.23	0.14	0.23
Number of medicines	68	56	62	52

There were some wide price variations for individual medicines. UNRWA prices were more than three times the price for a number of medicines including benzylpenicillin benzathine injection (20.9, 7.6 and 4.0 times the MSH, GCC and IDA price respectively), acetylsalicyclic acid 500 mg tablets (9.9 and 9.6 times the IDA and MSH price respectively), chlorphenamine 4 mg tablets (5.2, 4.6 and 3.3 times the MSH, IDA and GCC price respectively), and digoxin 0.25 mg tablets (3.4 times the MSH price). Conversely, UNRWA prices were lower than comparator prices for some medicines such as miconazole 2% oral gel (92% lower than the MSH price), salbutamol 0.5% respiratory solution (90% and 69% lower than the MSH and IDA price respectively), and indomethacin 100 mg suppositories (85% and75% below the GCC and MSH price respectively).

High prices of individual medicines compared to comparator prices indicates there may be opportunities for UNRWA to buy at lower prices and hence reduce expenditure. Taking into account the quantities of individual medicines procured by UNRWA in 2010, buying all or selected medicines at JPD, GCC and IDA prices was considered. If UNRWA were able to join the JPD tender or GCC tender, overall expenditure would increase by approximately US$360,000 (for 56 medicines) and US$ 1.88 million (for 62 medicines) respectively assuming no reduction in tender prices due to the increased quantity procured. Buying the 52 matched medicines from IDA would increase expenditure by approximately US$125,000. Competitive negotiation or joining the JPD or GCC tender for a limited list of medicines could be considered. As shown in Table 4, human 30/70 insulin offered the greatest savings if procured at the GCC price ($467,624), followed by amoxicillin + clavulanic acid tablets, benzlpenicillin benzathine vials, gliclazide and azithromycin suspension. If these five medicines could be procured at the lowest comparator prices, UNRWA would reduce annual expenditure by $1.4 million.

**Table 4:** **Medicines offering the greatest potential savings (to UNRWA central tender prices)**

Medicine	Therapeutic group	Comparator	Potential annual savings in USD
Neutral sol./isophane insulin 30/70 100 IU/ml 10 ml vial	Antidiabetic	GCC price	$ 467,624
Amoxicillin + clavulanic acid 500 + 125 mg tab	Antimicrobial	GCC price	$ 393,109
Benzylpenicillin benzathine 1/1.2MIU vial	Antimicrobial	GCC price	$ 243,822
Gliclazide 80 mg tab	Antidiabetic	JPD price	$ 210,907
Azithromycin 200 mg/5 ml suspension	Antimicrobial	JPD price	$ 144,489
*Total*			$ 1,459,951 (9.5% of General Fund expenditure on medicines)

An analysis of prices of medicines procured in 2010 via UNRWA’s central tender (centrally) and directly by each field (locally) showed that overall Syria was paying 20% less when procuring locally (30 medicines), Lebanon was paying 83% more (11 medicines), and West Bank was paying 128% more (18 medicines). Insufficient data was available for Jordan and Gaza to draw any conclusions.

## Discussion

In contrast to medicine price surveys undertaken using the World Health Organization (WHO)/Health Action International (HAI) methodology [9] in which a single comparator price (Management Sciences for Health) is used, this study utilized four reference prices. These were prices from a neighbouring and host country (Jordan’s Joint Procurement Department, JPD), a regional procurement organization (Gulf Cooperation Council, GCC), a non-profit international supplier (IDA Foundation, IDA), and an international price indicator guide (Management Sciences for Health, MSH) that consolidates prices from mostly non-profit suppliers and national government procurement bodies. By comparing to such a range of comparators we are able to ask the “what if” question in a more comprehensive way and a more detailed manner than if we had used a single comparator price.

Overall UNRWA was procuring medicines efficiently when the prices of centrally procured medicines were compared with MSH, JPD, GCC and IDA comparator prices. This is despite relatively small order quantities and the political challenges of procurement and delivery in this challenging environment.

An analysis by Cameron *et al.* showed that public sector procurement prices for 15 medicines (lowest-priced generics) in 39 predominantly low- and middle-income countries were, on average, 1.11 times MSH prices [10]. While most countries were procuring medicines at competitive prices, some countries were paying over three times MSH prices. In the WHO EMRO/HAI report of findings from nine countries in the Eastern Mediterranean region, median public sector procurement prices of lowest-priced generics where less than MSH prices in four countries (Jordan, Yemen, Pakistan and Jordan) and between 1 and 2 times MSH prices in five countries (Kuwait, Lebanon, Syria, Tunisia and Morocco) [4]. Our study showed that across a larger sample of 68 medicines, overall UNRWA was paying 0.99 times MSH prices hence achieving more competitive prices than many countries.

Based on the quantities procured by UNRWA, expenditure would increase if UNRWA procured the medicines in this analysis through the JPD or GCC tenders, or from IDA. However, savings are possible if some individual medicines were procured at lower comparator prices. We identified five medicines (one insulin, one oral antidiabetic medicine, and three beta-lactam antimicrobials) where substantial savings could be possible if they were procured at lowest comparator prices. In view of the heavy burden of antidiabetic medicines and antimicrobials on UNRWA medicine expenditure, pursuing options such as competitive negotiation with suppliers or joining the JPD and/or GCC tenders for these few medicines is recommended.

Another option would be to increase the number of UNRWA pre-qualified suppliers to attract more tender bids. At the time of the study, UNRWA had pre-qualified 101 suppliers, the vast majority of whom were from Europe and Jordan (few were from Asia). On average, three tender bids are received for each medicine. JPD receives an average of six bids per medicine, and the Jordan Food and Drug Authority has more than 500 suppliers of medicines (although the Jordan market is not as restricted as UNRWA’s). UNRWA should review the pre-qualified suppliers in Jordan and other host countries, as well as in other countries in the region, to identify potential new suppliers. Consideration should particularly be given to identifying potential suppliers from outside the region who supply quality-assured products and are likely to have competitive prices.

With budget constraints and a dynamic pharmaceuticals market, there is a need to regularly monitor prices and quantities in order to make informed decisions on the optimum procurement process, bids, contract termination etc. Sustainability is needed so limiting the review to the top 20 medicines by expenditure is recommended. Consideration of useful comparators is needed; they should be a mix of regional and international sources. Forging links with national medicine procurement departments should facilitate the data collection process. As well, the World Health Organization’s Regional Office for the Eastern Mediterranean should be encouraged to establish a regional procurement price database that UNRWA could both contribute to and access prices paid by countries in the region. Such a database has been developed in the World Health Organization’s Regional Office for the Western Pacific [11].

This study had a number of limitations including the limited number of comparators for the regional price comparisons. Prices were unsuccessfully sought from Lebanon, Syria and Egypt. No adjustments were made for additional freight charges and documentation costs often required for medicines supplied to Gaza and the West Bank. It must also be remembered that any savings are only potential. Competitive negotiation may not result in the prices of the comparators, and joining others tenders may not be possible. In addition, price is not the only consideration when procuring medicines. Processes are needed that ensure quantification is based on need, the medicines procured are of assured quality, and there are no interruptions in supply.

Policies that target preventable risk factors for diabetes, cardiovascular disease and other conditions are important to reduce morbidity and the need for pharmaceutical treatment. When medicines are needed, strategies to ensure they are appropriately prescribed and dispensed by health professionals, and appropriately used by patients, are also important.

## Conclusion

UNRWA is able to procure medicines in relatively small quantities at competitive prices demonstrating that irrespective of the challenges faced, good procurement practices result in prices that are close to international norms. With budget constraints and a dynamic market, there is a need to regularly monitor procurement prices and quantities in order to make informed decisions.

## Abbreviations

GCC: Gulf Cooperation Council
HAI: Health Action International
JPD: Jordan’s Joint Procurement Department
MSH: Management Sciences for Health
PHC: Primary Healthcare Centre
PIMS: Procurement Inventory Management System
UNRWA: United Nations Relief and Works Agency for Palestine Refugees in the Near East
USD: United States dollar
WHO: World Health Organization
WHO EMRO: World Health Organization Regional Office for the Eastern Mediterranean.
